# Human and Mouse Mononuclear Phagocyte Networks: A Tale of Two Species?

**DOI:** 10.3389/fimmu.2015.00330

**Published:** 2015-06-25

**Authors:** Gary Reynolds, Muzlifah Haniffa

**Affiliations:** ^1^Human Dendritic Cell Laboratory, Institute of Cellular Medicine, Newcastle University, Newcastle upon Tyne, UK; ^2^Musculoskeletal Research Group, Institute of Cellular Medicine, Newcastle University, Newcastle upon Tyne, UK

**Keywords:** mononuclear phagocyte system, dendritic cells, macrophages, monocytes, comparative genomics

## Abstract

Dendritic cells (DCs), monocytes, and macrophages are a heterogeneous population of mononuclear phagocytes that are involved in antigen processing and presentation to initiate and regulate immune responses to pathogens, vaccines, tumor, and tolerance to self. In addition to their afferent sentinel function, DCs and macrophages are also critical as effectors and coordinators of inflammation and homeostasis in peripheral tissues. Harnessing DCs and macrophages for therapeutic purposes has major implications for infectious disease, vaccination, transplantation, tolerance induction, inflammation, and cancer immunotherapy. There has been a paradigm shift in our understanding of the developmental origin and function of the cellular constituents of the mononuclear phagocyte system. Significant progress has been made in tandem in both human and mouse mononuclear phagocyte biology. This progress has been accelerated by comparative biology analysis between mouse and human, which has proved to be an exceptionally fruitful strategy to harmonize findings across species. Such analyses have provided unexpected insights and facilitated productive reciprocal and iterative processes to inform our understanding of human and mouse mononuclear phagocytes. In this review, we discuss the strategies, power, and utility of comparative biology approaches to integrate recent advances in human and mouse mononuclear phagocyte biology and its potential to drive forward clinical translation of this knowledge. We also present a functional framework on the parallel organization of human and mouse mononuclear phagocyte networks.

## Introduction

The mononuclear phagocyte system (MPS) is a branch of the immune system comprising dendritic cells (DCs), macrophages, and monocytes (1–3). The many functions of the MPS include tissue maintenance and healing, innate immunity and pathogen clearance, and the induction of adaptive immune responses (1–3). Manipulating these functions could lead to clinical benefit, such as modulating DCs to develop antigen-specific anti-tumor immunity or suppressing peripheral autoreactive T cell responses in autoimmunity (4, 5). Several factors need to be considered in designing immunotherapy targeting the MPS, including cellular or pathway target choice and the relevant disease and tissue context. Diversity and plasticity of the MPS, two core features that are paramount for directing the quantity and quality of specific immune responses, have frustrated attempts to develop successful focused therapies. The additional variable of local tissue environment, which also heavily influences the composition and function of resident and infiltrating mononuclear phagocytes (MPs), also requires careful consideration (1–3).

The MPS was conceived in the 1960s by van Furth to encompass a family of phagocytic mononuclear leukocytes regarded as functional variations of monocytes (6). DCs were embraced as members of the MPS several years later (7). The revolutionary discovery that human monocytes and CD34^+^ hematopoietic stem cells (HSCs) could be differentiated into DC (mo-DC) and macrophage-like (mo-Mac) cells provided a convenient *in vitro* model to study human MP biology (8–10). However, murine studies have demonstrated the independence of many DCs, macrophages, and Langerhans cells (LCs) from blood monocytes questioning the accuracy of human *in vitro* monocyte-derived cells in recapitulating *in vivo* populations (11–16). Conventional DCs arise from HSCs along a lineage that does not go through a monocyte stage and are dependent on the growth factor receptor FLT3 (11). In contrast, the majority of tissue macrophages arise from prenatally seeded precursors that can survive into adulthood and are dependent on CSF1-R (12–16).

**Figure 1:**
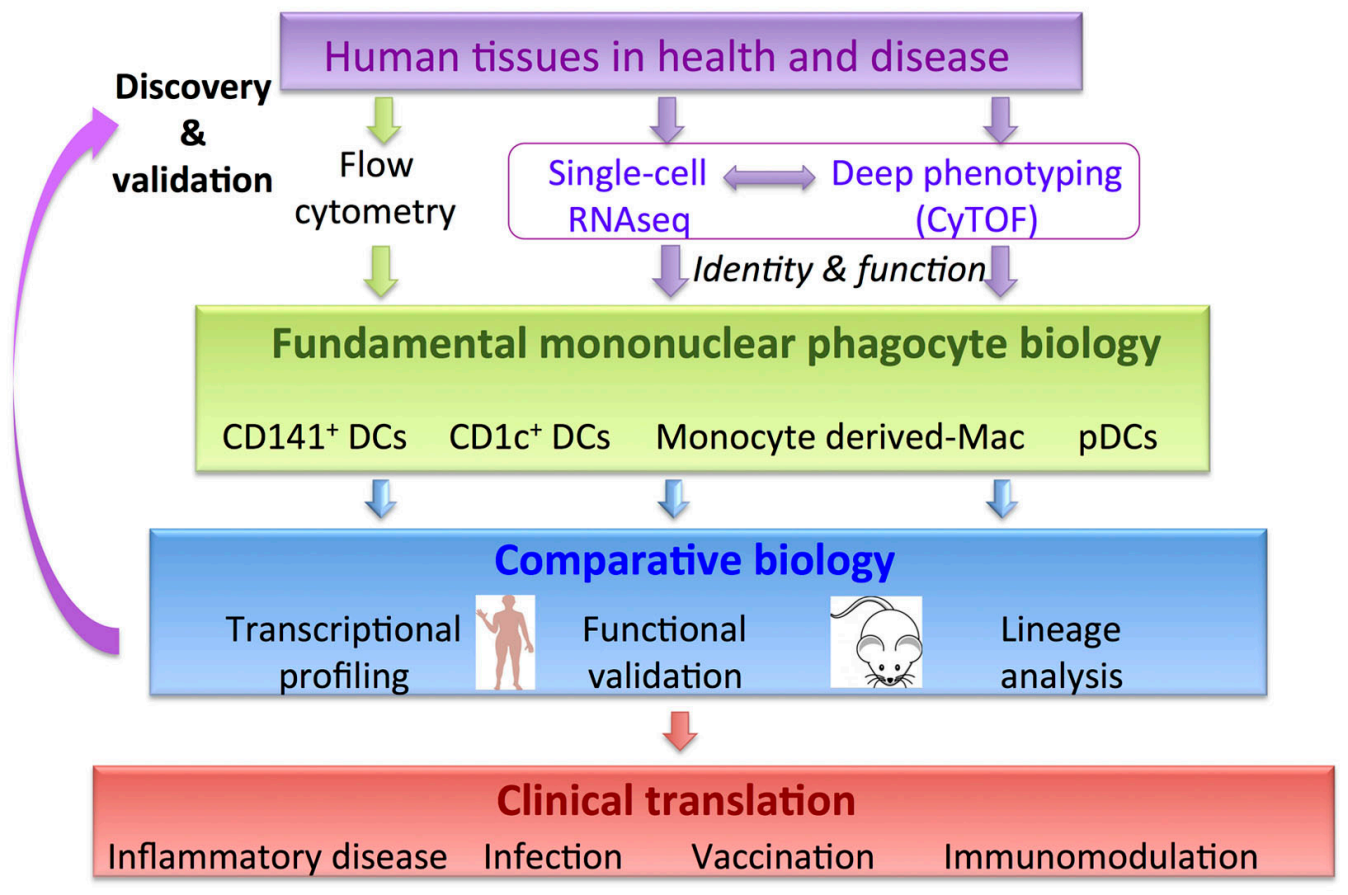
**Comparative biology is a validation and discovery tool to pull-through fundamental knowledge in MPS biology to clinical translation**. Incorporation of new genomics and proteomics methodologies will accelerate discovery.

The constituents of MPS share overlapping surface markers, which poses a challenge in parsing functionally distinct populations. A rewarding approach to unravel this complexity has been comparative biology analysis (17–28). In essence, comparative biology relies on the concept that core developmental programs and functions such as differential CD4 and CD8 T cell priming, cross-presentation, migration, and cytokine production are likely to be non-redundant and conserved between species. In support of this, around 99% of murine genes have human analogs and around 96% are syntenic, despite the two species having 80 million years of divergent evolution (29). Comparative transcriptomic mapping has revealed conserved gene expression profiles in the two species allowing parallels to be drawn between DC and macrophage subsets (17–28). This approach places comparative analysis as the central fulcrum facilitating the integration of fundamental immunology to fertilize clinical translational strands (Figure 1). Integrating this workflow with cutting-edge technologies including single-cell genomics and proteomics approaches has the potential to accelerate discovery in basic MP biology and its clinical applicability (Figure 1). Comparative biology has revealed further insights into the origin and function of human and mouse mononuclear phagocyte populations (17–28) and generated new hypotheses to be tested in both species.

**Figure 2:**
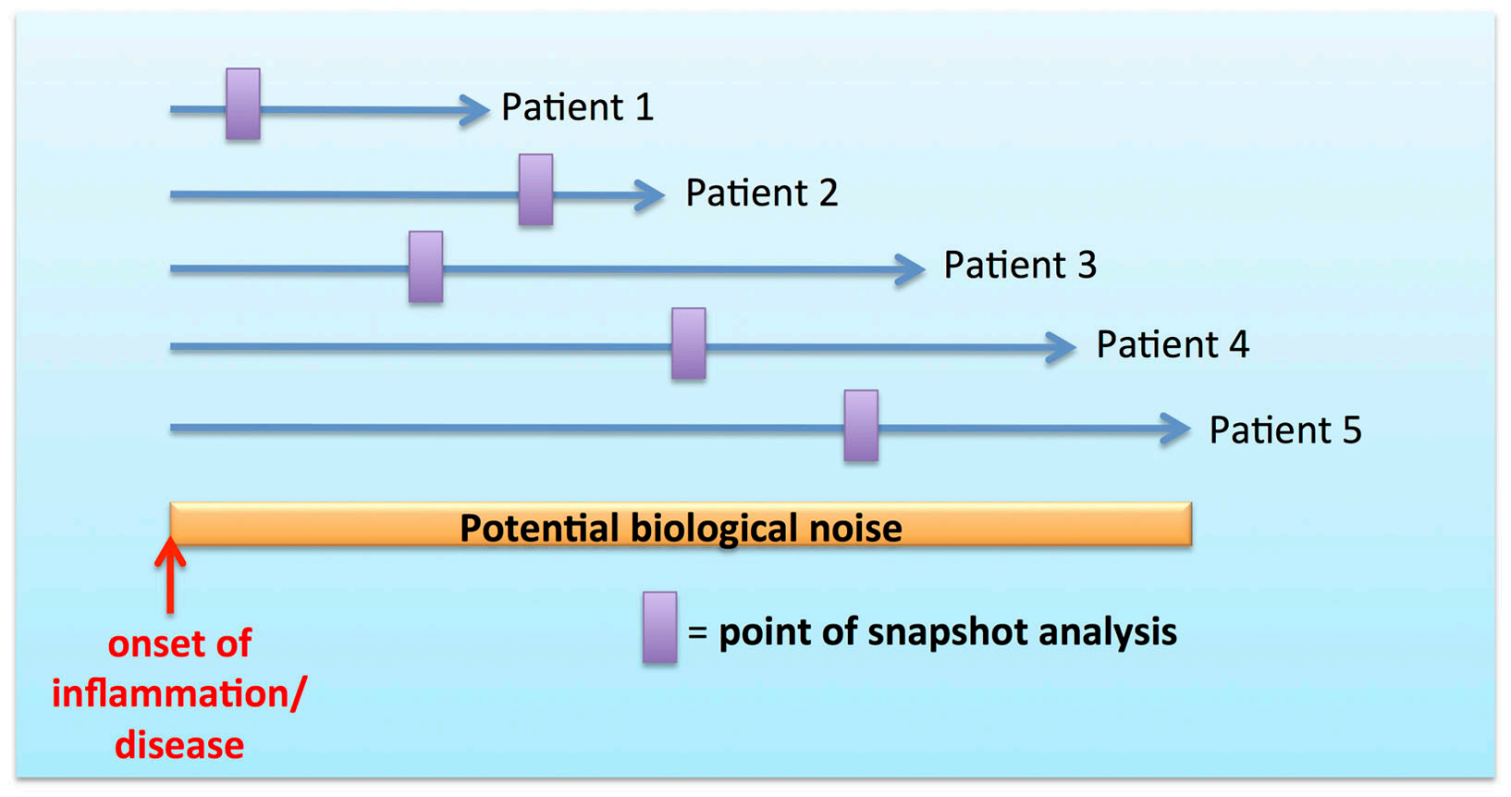
**Biological noise with snapshot analysis during temporal course of inflammation and disease**.

The concept of functional specialization as an inherent property imprinted by MP ontogeny and tissue anatomy has been well demonstrated in many murine studies [reviewed in Ref. (1, 3, 30)]. However, the MPS possesses an additional layer of complexity in the form of dynamic mobility, plasticity, and adaptability to tissue/local microenvironment both in steady state and in inflammation (1, 3, 31). These issues have been particularly difficult to dissect in human, where the temporal resolution to observe these kinetics is constrained by snapshot analysis during inflammation and disease without adequate recourse to their onset and evolution (Figure 2). Snapshot observations during inflammation may be confounded by temporal variations in MPS composition and function resulting in highly variable biological data. This variability may account for the biological noise inherently observed with outbred humans in contrast to inbred mice in specific pathogen free (SPF) facilities.

Mononuclear phagocytes and their progenitors are in dynamic equilibrium between peripheral tissue, blood, and bone marrow (1, 3, 31, 32). The distinction between MPs within peripheral interstitial tissue and blood can be difficult to establish in highly vascularized organs such as liver and spleen, where large sinusoids are present adjacent to discontinuous endothelial lining that enables greater mobility of leukocytes within these organs. In addition, inflammatory perturbations affect the dynamic equilibrium between tissue, blood, and bone marrow compartments favoring the relative expansion and egress of specific lineages in response to distinct stimuli (33–35). Expansion of monocyte-derived cells dominates the response to inflammatory stimuli in tissue but little is known regarding their fate upon resolution of inflammation (35). Peripheral tissue DCs migrate to the lymph node where they mediate their potent functions upon inflammatory stimuli. Whether they play a prominent role in local tissue immune regulation and how migratory DCs are repopulated during inflammation and its resolution has been poorly characterized.

## Comparative Biology to Interrogate Human and Mouse MP Networks

Identifying homology between mice and humans in other hematopoietic cells such as T and B cells has been relatively simple at phenotype and practical levels because of shared lymphocyte surface markers (CD3/CD4/CD8 and CD19, respectively) as well as the relative ease of isolating lymphocytes, which form 90% of human peripheral blood mononuclear cells (c.f. <1% being DCs). Nevertheless, there are functional differences in lymphocytes between the two species, such as differentiation requirements for IL-17 (36) and GM-CSF (37, 38) secreting CD4^+^ T cells, the specificity of granzyme and FOXP3 expression to define natural Tregs (39), the distinct classes of immunoglobulin (40) and human CD1a, 1b, and 1c-restricted responses to lipid molecules (41). Unfortunately, components of the human and mouse MPS lack overlapping phenotypic markers, hampering initial progress in identifying homologous populations between species.

A range of –omics technologies such as transcriptomics, metabolomics, proteomics, and epigenomics could potentially be employed to assess proximity between species. Of these approaches, transcriptomics is technically most tractable and generates enough complexity to achieve good definition between populations (*n*-dimensions where *n* is the number of genes analyzed) (42, 43). Transcriptome-based comparison of various hematopoietic lineages between human and mouse shows broad conservation but also highlighted specific differences and transcriptional divergence due to gene duplication (43).

### Transcriptomics

The hypothesis underlying comparative transcriptomics is that the identified MP populations were present in a shared ancestor and that these same subsets are present in modern animals. Furthermore, despite divergent evolution over time, cells from each subset will have a conserved transcriptomic signature similar to that of its equivalent in the other species. Two approaches are generally used to measuring this similarity: (1) unsupervised hierarchical clustering and principal component analysis (PCA), which assigns samples a point in *n*-dimensional space (*n* corresponding to the number of genes analyzed) and applying a distance metric with greater proximity suggesting a developmental relationship, or (2) supervised assessment of defined transcriptome signature enrichment between populations of interest exemplified by gene set enrichment analysis (GSEA) (44) and its later variations (45).

In hierarchical clustering, the Euclidean distance is calculated between samples. In PCA, the same Euclidean metric is used after the *n*-dimensional data are projected on to the two or three dimensions over which the most variation occurs. This approach has the disadvantages inherent in using large sets of gene data, large number of variables/genes, and high inter-sample variability when testing a limited number of samples. The consistent finding that tissue-specific genes predominate in DC microarray transcriptomes highlights the first point. As a result, microarray data of DC subsets from the same tissue tend to cluster together rather than with their equivalent in blood or another tissue (46). This can be corrected for by techniques such as excluding genes that are differentially expressed between pooled cells from each tissue (and classifying these “tissue-specific”) (23, 26) or through using an abbreviated gene panel that is enriched for genes that are known to give good definition between DC subsets (17). An important corollary of this finding is that, while the relative contribution of ontogeny and environment to DC function remains to be determined, the list of genes that define ontogeny is a small fraction of the genes that are modulated by the environment and highlights a potential drawback of using blood DCs as a proxy for tissue DCs.

**Figure 3:**
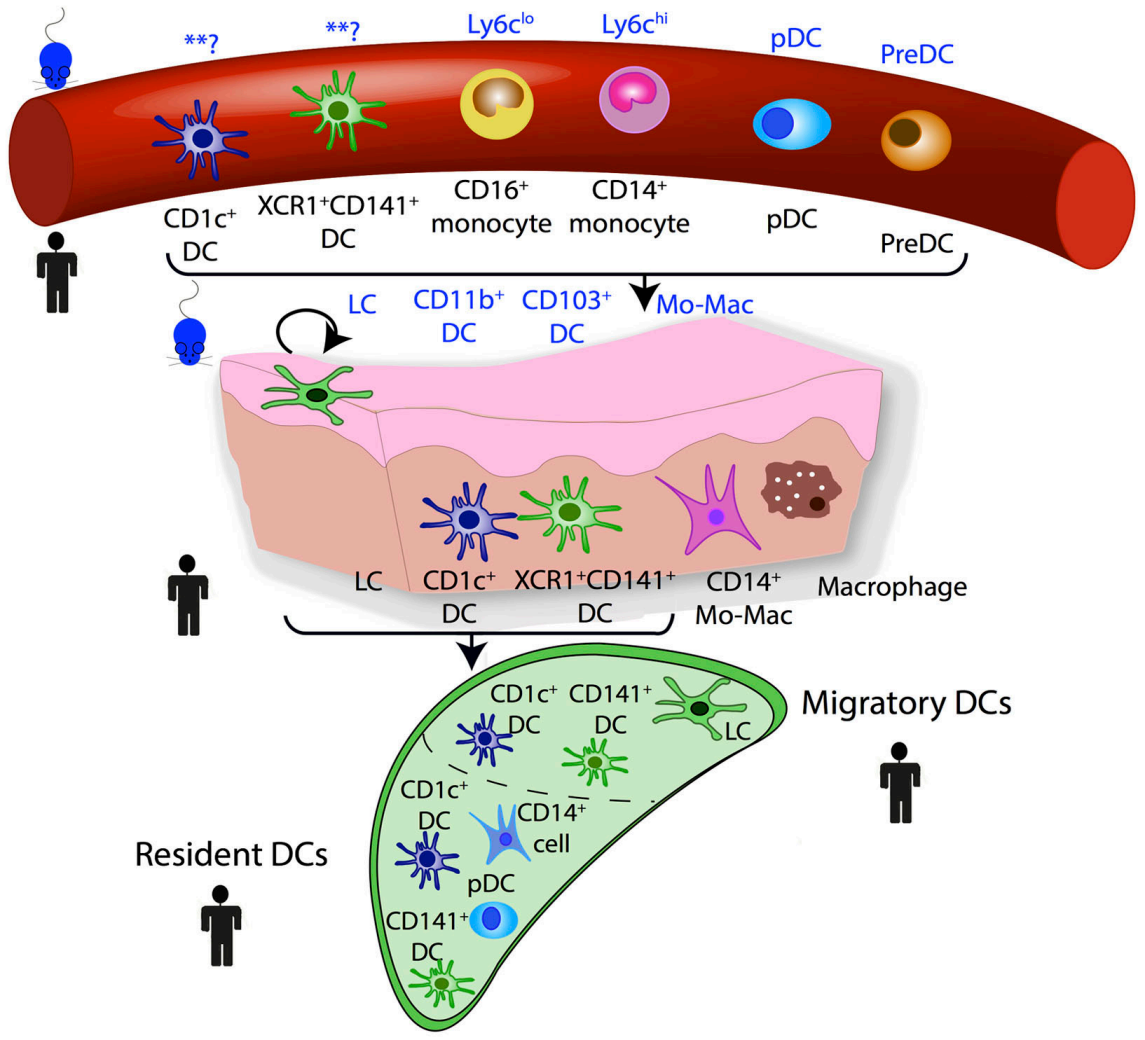
**Human and mouse mononuclear phagocyte networks**. **? denotes uncertain murine homologs.

The use of GSEA derives from large-scale microarray data in which it was recognized that groups of co-regulated functionally linked genes may be more relevant than the few genes that are most significantly differentially regulated but functionally unrelated. This approach is dependent upon an *a priori* understanding of gene function and this can introduce bias. When GSEA has been used in aligning DC subsets between species, a “query signature” is produced that defines the subset of interest. Samples in the test population can then be interrogated for whether they are enriched for this query signature. The underlying analysis is based on the non-parametric goodness-of-fit Kolmogorov–Smirnov test statistic with the reference probability distribution that of the query signature. GSEA and its later variant connectivity map analysis (CMAP) have been successfully used to identify homologous MP populations between species and the developmental origin of human inflammatory DCs (17, 23, 25, 26, 47). Steady state homologous MP populations in human and mouse blood, lymph node, and peripheral tissues are illustrated in Figure 3.

Most transcriptomics studies thus far on MPs have involved ensemble or bulk-population analysis. This introduces an inherent bias, as cell populations have to be defined *a priori* based on expression of specific markers. More recently, the application of single-cell RNA-sequencing (sc-RNA-Seq) with unbiased analysis potential has been successfully used to interrogate cellular heterogeneity to uncover new cell populations, functional immune states, and to establish cellular lineage hierarchies and lymphocyte differentiation programs (48–53). These technical advances combined with novel computational approaches have the potential to revolutionize our understanding of MPS biology by unraveling predicted and unexpected functional heterogeneity, which underpins the dynamic repertoire of our immune system in health and disease.

### Proteomics

Proteomics analysis has revealed differences in viral sensing pathways between murine splenic DC subsets (54) and identified the murine common monocyte progenitor (cMOP), an intermediate cell-type between the monocyte/macrophage and DC precursor (MDP) and monocyte (55). However, current large-scale proteomics approaches require high cell numbers for robust analysis and are impractical for rare populations, especially from limited human tissue material. Protein expression on a more limited scale has been the mainstay of conventional flow cytometry to define populations and assess MP functions at single-cell resolution. Although the number of parameters that can be analyzed simultaneously is limited (17–18 parameters using commercial instruments), the application of new unbiased probabilistic analysis to define populations could reveal new insights to MP heterogeneity (56). Mass cytometry (CyTOF) provides additional parameters (up to 100) and combined with unbiased population assignment has enormous discovery potential. This combined analysis on mouse myeloid cell populations has revealed far greater population heterogeneity than previously appreciated (57).

### Functional validation

**Figure 4:**
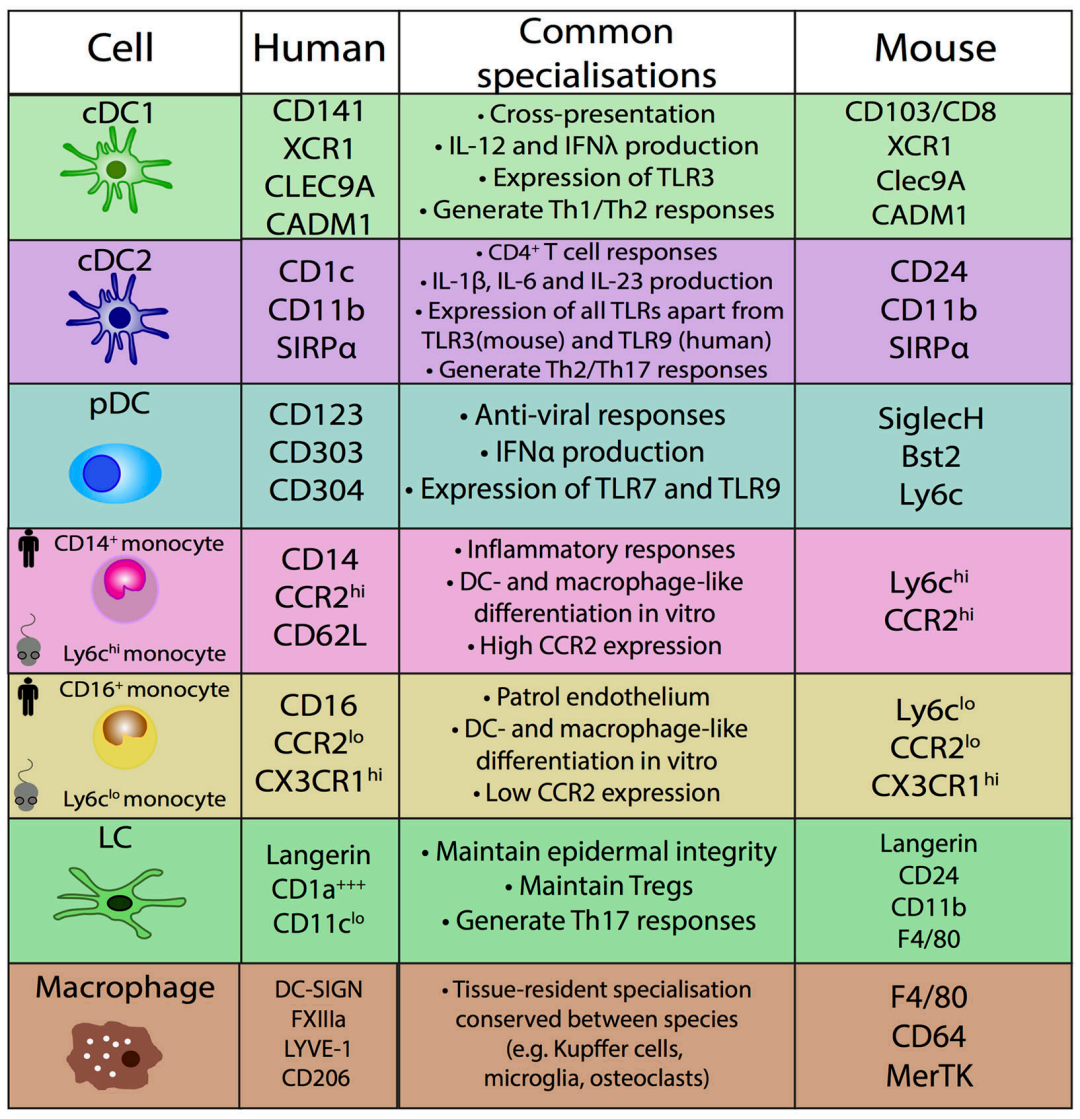
**Conserved specializations between human and mouse mononuclear phagocytes**.

Comparative functional analysis between mouse and human MPs has resulted in variable findings [reviewed in Ref. (30, 58)]. It is unknown if this is due to true biological differences or experimental factors which are not comparable within and between species, including the common use of murine *in vivo* models in contrast to human *in vitro* assays to assess MP functions. Conserved functions are detailed in Figure 4.

### Lineage analysis

The power and utility of comparative biology to identify homologous MP populations is beginning to be applied to MP lineage analysis. The recent identification of the successive downstream progenies of human MDP; the Common DC precursor (CDP) and precursor of myeloid DCs (pre-cDCs) exploited the conserved dependency on growth factors and cytokines between human and mouse DC precursors (27, 28). Similarly, comparative analysis suggested the monocyte-origin of human dermal CD14^+^ cells (25) and inflammatory DCs (47). The preservation of LCs and dermal macrophages in GATA2 and biallelic IRF8 deficiencies show that they are bone marrow independent in the steady state and similar to their murine counterparts, also arise from prenatally seeded precursors (59, 60).

## DC, Monocyte, and Macrophage Subsets in Mice and Humans

This approach of using ontogeny and by extension transcription factor dependence to define MPS populations was formalized recently in a proposed nomenclature (61). In this scheme, four adult HSC-derived MP populations are described in mice: two conventional/classical DC subsets (cDC1 and cDC2), plasmacytoid DCs (pDCs), and monocyte-derived cells (61). Both cDCs and pDCs are derived from murine CDP (62, 63). The CDP-derived cells are defined by their dependence on specific transcription factors (TFs): cDC1 are Batf3-dependent, cDC2 are Irf4-dependent, and pDC are E2-2-dependent (61). This definition is unambiguous and avoids using surface markers that can vary between tissues and in inflammation. While the ontogeny approach aids definition of murine populations, it cannot be easily transferred to human DC nomenclature, due to inherent logistical difficulties of human ontogeny studies. However, with the aid of comparative biology approaches, homologous populations between human and mouse MP subsets can be identified and inferences between species on ontogeny and function can be made (Figures 3 and 4).

### cDC1

#### Phenotype

This subset is identified in mouse by the expression of CD8α in the spleen and CD103 in non-lymphoid tissues (NLT). Its human equivalent in blood and NLT were initially defined by their high expression of CD141 (thrombomodulin, BDCA-3) (19–23). However, this antigen can be upregulated on blood monocytes and expressed promiscuously by other DC subsets in human tissue (23). The cell adhesion molecule CADM1 (NECL2), C-type lectin, CLEC9A (which recognizes damaged cells), and the chemokine receptor XCR1 are expressed on human and mouse cDC1 (19, 22, 64–66). However, CADM1 expression is not restricted to leukocytes and CLEC9A is also expressed on murine DC precursors (67, 68). Although cDC1 is the only leukocyte expressing XCR1, a commercial antibody against it is currently unavailable. Notably, langerin is expressed on murine but not human cDC1 (23, 69, 70).

#### Homology

Homology between human (XCR1^+^CD141^+^DCs) and mouse (CD8^+^/CD103^+^) cDC1 was demonstrated by comparative transcriptomics, phenotype, and functional analyses (17, 19–21, 23, 71). Furthermore, blood and skin CD141^+^ DCs cluster together separately from CD1c^+^ DCs, CD14^+^ and CD16^+^ monocytes, and pDCs, suggesting that skin XCR1^+^CD141^+^ DCs are the tissue equivalents of blood XCR1^+^CD141^+^ DCs (23).

#### Transcription Factors

In addition to Batf3 (72), murine cDC1 differentiation requires Irf8 (73), Id2 (74, 75), and NFIL3 (76). In human, shRNA knockdown of *BATF3* in cord blood HSCs inhibits their differentiation into cDC1 *in vitro* (22). However, cDC1 were detectable in humanized mice reconstituted with *BATF3* knockdown CD34^+^ HSCs (22). A possible explanation for this seeming contradiction was shown in mice, where in inflammatory conditions (specifically in the presence of IL-12), other members of the Batf family of TFs appear to be able to compensate for loss of Batf3 (77). *ID2* mRNA is expressed at low amounts in human CD34^+^ HSCs but upregulated during DC differentiation in the presence of GM-CSF and IL-4 (74). Its role is potentially in suppressing B cell differentiation from a common precursor. Definitive evidence for the requirement of ID2 and NFIL3 in cDC1 development in humans is lacking and highlights the potential difficulties of translating TF-based definitions of DC subsets from mice to humans.

#### Function

The cDC1 subset is thought to be able to efficiently prime CD8^+^ T cells through functional specializations such as cross-presentation of antigens and the production of IL-12p70 (78–80). This process is important in the induction of tumor immunity and the control of viral and bacterial infections when DCs are not the malignant cells or directly infected. The expression of Clec9A and XCR1 by both murine and human cDC1s supports this notion. cDC1s express a more limited TLR profile than cDC2s with high expression of TLR3 and TLR10 but without TLR4, –5, –7 and –9 (54, 81). TLR3 senses viral dsRNA but the role of TLR10 is currently unknown. Human cDC1s do not produce large amounts of IL-12p70 in response to TLR ligands alone but do following the combination of TLR ligands and CD40-CD40L signaling through activated T cells (71), in common with the finding in mice (82). IFN-λ is produced by murine and human cDC1 upon stimulation with the TLR3 agonist, poly I:C (83).

Murine cDC1s have an advantage over other subsets at cross-presentation of antigens by being able to (1) maintain optimal phagosomal pH for antigen processing (84) and (2) enhance the transfer of proteins from the endosome in to the cytosol so they can be loaded on to MHC Class I (85). This advantage is apparent when assessing cross-presentation of dead cell-derived antigens and upon stimulation with TLR3. However, recent data showed that murine cDC2 are also able to cross-present and cross-prime antigen upon stimulation with R848, a TLR7/8 agonist (86). In human, cDC1 appears to be superior at cross-presenting cell-derived antigen, particularly upon polyI:C stimulation (19–21, 71) and when antigens are delivered to late endosomes and lysosomes (87). However, in common with mice, cDC2 are also able to cross-present soluble antigen and long-peptide particularly upon R848 stimulation (88, 89). The variable findings reported may also be due to type of antigens used in the cross-presentation assays and the validity of comparing murine *in vivo* models with human *in vitro* assays.

In mouse, cDC1 preferentially induce Th1 immune response through IL-12p70 production (90, 91), although Th2 induction has also been reported (92). In human, both cDC1 and cDC2 have been shown to induce Th1 and Th2 responses (93). cDC1s were also shown to promote enhanced Th2 differentiation in response to TSLP in an influenza infection humanized mouse model (94). As most human experiments are performed using blood DCs and *in vitro*, it has been logistically difficult to establish pathogen and tissue-specific effects relevant for driving Th priming *in vivo*.

### cDC2

#### Phenotype

cDC2s in mice are lin^–^MHCII^hi^CD11c^+^CD11b^+^. However, this fraction also includes monocyte-derived cells and macrophages (95). This is demonstrated by the variable depletion of cells from this fraction in Flt3 or Csf1r KO mice suggesting contamination by Flt3-independent cells (75). This is in contrast to the near complete absence of cDC1 in Flt3 KO mice (74).

Genetic tracing using Clec9A-reporter mouse to identify all CDP-derived cells demonstrated near-complete labeling of cDC1s but variable labeling of CD11b^+^ DCs in NLT (68). Although this is in keeping with the presence of monocyte-derived cells and macrophages within CD11b^+^ cells, it does not exclude the possibility of an alternative DC differentiation program that does not undergo a monocyte or CDP intermediate stage. Splenic CD11b^+^ DCs are divided into an ESAM^hi^ population that requires Notch2-, Flt3, and LTβ-signaling for its development and a monocyte-like ESAM^lo^Clec12A^+^CX3CR1^+^ population that is Flt3-independent and expresses high levels of CD14, TNFα, CCR2, and Lyz2 (96). In murine lung, it has been possible to divide the MHCII^+^CD11c^+^CD11b^+^ fraction into CD11b^+^CD64^+^ monocyte-macrophage cells and CD11b^+^CD24^+^ cDC2s (24). In murine skin, the MHCII^+^CD11b^+^Langerin^–^ fraction comprises cDC2, monocyte-derived cells, and macrophages (97).

There is evidence that similar heterogeneity may be present within human cDC2. Only 170 genes characterized human cDC2, in comparison to 1020 for cDC1 and 1065 genes for pDCs (23). This limited list of differentially expressed genes predicts heterogeneity within the boundaries of the phenotype parameters used to define human cDC2, specifically a subpopulation derived from or closely related to another mononuclear phagocyte such as CD14^+^ monocytes.

Human cDC2 (CD1c^+^ DCs) are defined as lin^–^MHCII^+^CD14^–^CD16^–^CD11c^+^CD1c^+^ cells, a definition they share with *in vitro* monocyte-derived DCs. Although human peripheral blood and murine cDC2 additionally express CD11b, CX3CR1, and SIRPα, these antigens do not distinguish them from monocyte-derived cells (24, 98). Uniquely in the small intestine, cDC2s co-express CD103 and SIRPα (24, 26). *In vitro* human mo-DCs express CD206/MMR and CD1a but peripheral blood cDC2 do not (47, 99). However, tissue CD1c^+^ DCs express CD206 and CD1a (100, 101). In addition, some tissue CD1c^+^ DCs co-express CD14 particularly during inflammation (47).

#### Homology

The transcriptional signatures of human blood CD1c^+^ DCs are enriched with that of mouse spleen CD4^+^/CD11b^+^ DCs (17, 23). In NLT, the transcriptional signatures of human small intestine CD103^+^SIRPα^+^ DCs and dermal CD1c^+^ DCs are enriched with that of murine spleen and mesenteric lymph node CD11b^+^ DCs and dermal CD11b^+^ DCs, respectively (25, 26). A similar relationship was also observed between murine lung CD11b^+^ DCs with human blood CD1c^+^ DCs (24).

#### Transcription Factors

cDC2 development has been shown to be dependent on the TFs Irf4, PU.1, RelB, and RBPJ (24, 96, 102–108). Irf4 directly supports MHC class II antigen presentation to promote CD4^+^ T cell responses (109). In humans, CD1c^+^ DCs express high amounts of IRF4 (24). Interestingly, IRF4 is also required for mo-DC differentiation, suggesting a shared differentiation program between cDC2 and mo-DC. PU.1 interacts with Irf4 but also upregulates Flt3 expression critical for early DC differentiation in mice (110, 111). The PU.1 binding site in the Flt3 promoter is conserved in mice and humans, and so it is thought to be similarly required for DC differentiation in humans (111). Administration of Flt3 results in expansion of DC subsets in lymphoid and non-lymphoid tissue (112). PU.1 mutations in humans and mice are associated with myeloid leukemias(113). Biallelic human IRF8 K108E mutation resulted in complete loss of monocytes, pDCs, cDC1, and cDC2 in the peripheral blood (60). Surprisingly, human autosomal dominant IRF8 T108A mutation results in selective loss of the cDC2 subset and IL-12 production (60). It is now apparent from studies on Irf8^R294C^(BXH2) and Irf8^-/-^ mice that in addition to cDC1, pDCs and monocytes are also dependent on IRF8 (73, 75, 114–116). However, cDC2 frequency in mice with Irf8^-/-^ and the hypomorphic mutation Irf8^R294C^ are unaffected, in contrast to the findings in humans (73, 75, 114).

#### Function

The transcriptome of cDC2s is enriched for genes related to antigen processing such as LAMP1, LAMP2, and cathepsins (117). Murine cDC2s have been shown to be able to promote Th17, Th2, and regulatory T cell responses depending upon the pathogen and antigen stimulus (24, 108, 118–120). This may be a consequence of their innate plasticity but could also relate to unresolved heterogeneity within murine cDC2. In human, cDC2 have been shown to induce Th17 differentiation (24).

Both human and mouse cDC2 share many transcriptional and functional similarities with monocyte-derived cells (24, 25, 47, 97). Both cDC2 and mo-DC are capable of promoting naïve CD4^+^ and CD8^+^ T cell proliferation and in mice cDC2 appear to be superior at trafficking to lymph nodes (97, 98), leading to the hypothesis that mo-DCs specialize in activating tissue-tropic T cells. Mo-DCs also produce higher levels of monocyte-attracting chemokines (CCL2, CCL7, CCL12) than cDC2s (98). Human blood cDC2 have a TLR expression profile that is close to murine lymphoid cDC2 with significantly higher levels of TLRs 2, 4, and 5 than other DC subsets (81), a profile it also shares with *in vitro* mo-DCs (121). The pathogenic role of cDC2 in human disease is not clear but they have been shown to accumulate in conditions such as RA (122), chronic kidney disease (123), and atopic airway inflammation (124), although their distinction from inflammatory mo-DCs is unclear. Human cDC2 are also implicated in the accumulation of CD103^+^CD8^+^ mucosal T cells in the lung and promote fibrosis in the kidney through production of TGFβ (123, 125). Finally, human and mouse cDC2 share a similar cytokine production profile which includes IL-6, IL-23, and IL-1β (24, 81, 126, 127). In addition, unlike murine cDC2, human blood cDC2 can secrete high amounts of IL-12p70 upon in vitro stimulation with R848 and LPS, which was augmented in the presence of IFNγ and CD40L (89).

### Plasmacytoid DCs (pDCs)

#### Phenotype

Plasmacytoid DCs are specialized IFNα producing cells that were first described in human peripheral blood and tonsil (128–131). In blood, their morphology resembles that of lymphocytes but upon *in vitro* culture with IL-3 and CD40L, they acquire dendrites resembling myeloid DCs (129). pDCs are identified in mice by expression of CD11c^int^CD11b^–^B220^+^ in combination with markers such as SiglecH and CD317 (BST2) to exclude a subset of NK cells and precursors of cDCs (132). In humans, they are identified by expression of CD123, CD303, and CD304. CD123 is the IL-3 receptor alpha chain and is also expressed on precursor cells, basophils, and eosinophils (133, 134). CD303 (BDCA-2) is a C-type lectin that is specifically expressed by human pDCs (135). Functionally, it has a role in antigen capture and when ligated it inhibits IFNα production (136). CD304 (BDCA-4) is uniquely expressed by pDC in peripheral blood but is also expressed by other cells such as endothelial cells (137).

#### Homology

The relative distance of the pDC transcriptome from other leukocyte subsets and its conservation directly aligns murine and human pDCs (17). However, a subset of murine pDCs also appears to have cDC differentiation potential (138, 139), which has not been observed in human.

#### Transcription Factors

Plasmacytoid DC development in humans and mice is dependent on the transcription factor E2-2 (140). E2-2 opposes default differentiation of precursors into cDCs and controls expression of a range of pDC-associated TFs, including SpiB, Irf7, and Irf8 (140, 141). In humans, haploinsufficiency of E2-2 results in Pitt-Hopkins syndrome, a condition with a range of features including developmental delay and characteristic facial features but without known clinical immunodeficiency (142). A population of CD45RA^+^CD123^+^ cells is present in the blood of patients with Pitt-Hopkins syndrome but these cells fail to express CD303 and have severely reduced expression of IFNα, indicating that loss of E2-2 blocks full pDC differentiation (140). The transcription factor SpiB is required for IFNα production by pDCs in mice (143). SpiB-knockdown in human CD34^+^ HSCs inhibits pDC differentiation *in vitro* (144).

#### Function

Plasmacytoid DCs have a functional program that is well-conserved between mice and humans (145). In contrast to cDCs, pDCs express a narrow range of pattern recognition (146). Both mouse and human pDCs express TLR7 and TLR9 (146). TLR8 is expressed at very low amounts if any by human pDCs (81, 147, 148) and appears to have a different function in mice (146, 149, 150). pDCs in both mice and humans are specialized in the production of IFNα and thought to be important in viral immunity but also human autoimmunity such as SLE (151, 152).

### Monocytes and monocyte-derived cells

#### Phenotype

Two subsets of monocytes exist in mice and can be distinguished by the differential expression of Ly6C, CCR2, and CX3CR1. Similarly in humans, there are two monocyte subsets in peripheral blood identified by expression of CD14 and CD16 (CD14^++^CD16^–^ and CD14^+^CD16^+^) as well as an intermediate phenotype (CD14^++^CD16^+^). In addition to these antigens, human monocytes are also heterogeneous for the expression of the angiopoietin receptor, Tie2, and 6-sulfoLanNAc(Slan), a carbohydrate modification of the P-selectin glycoprotein ligand-1 (PSGL-1) (153, 154).

#### Homology

Homology between peripheral blood monocyte subsets has been demonstrated by the extensive transcript enrichment between Ly6C^hi^CX3CR1^lo^ and CD14^++^CD16^-^ monocytes and between Ly6C^lo^CX3CR1^hi^ and CD16^+^ monocytes (18, 23, 155).

#### Transcription Factors

The TFs that regulate the sequential differentiation of HSCs into MDP in mice include PU.1, Irf8, and Klf4 [reviewed in Ref. (156)]. PU.1 is required at each developmental bifurcation including HSC maintenance (157) and the generation of early myeloid progenitors (16, 158–160). Similarly in humans, PU.1 is required for monocyte differentiation from CD34^+^ cord blood precursors (161). In murine monopoiesis, Irf8 and Klf4 act together to skew differentiation toward monocytes by antagonizing the granulocyte-supporting TF C/EBPα (115, 162). Consistent with this, human autosomal recessive Irf8 deficiency results in complete loss of circulating monocytes and DCs in the presence of neutrophilia (60).

The TFs that control cell-fate decisions downstream of MDP are less well defined. In mice, Irf5 and TCFEB are implicated during MDP to CMoP differentiation (55). The TF Nur77 has been implicated in Ly6C^lo^CX3CR1^hi^ monocyte generation (163).

PU.1 and MafB act antagonistically to support human monocyte differentiation into mo-DC and mo-Mac, respectively *in vitro* (164). Irf4 was also implicated in human *in vitro* mo-DC differentiation (165). Irf5 promotes the differentiation of classical/M1 macrophages from human monocytes *in vitro* (166). In contrast, Irf4 activates transcription of the alternative/M2 macrophage markers in mice (167) and humans (168, 169).

#### Function

CD14^+^ human and Ly6C^hi^CX3CR1^lo^ murine monocytes can exhibit considerable functional plasticity as demonstrated by their acquisition of DC-like and macrophage-like characteristics *in vitro* and *in vivo*. Recent fate mapping studies have demonstrated that monocytes do not contribute to tissue-resident macrophages in the steady state (12, 14, 15), with the notable exception of gut and dermal macrophages (14, 97, 170). However, monocytes can give rise to tissue macrophage-like cells in inflammation (35, 171). Monocytes can also differentiate into DC-like cells in the steady state in mucosal tissues and skin (97, 172). This process is enhanced during inflammation (97, 98, 173), including infections with Leishmania (34), Influenza (174), Trypanosoma (175), Listeria (33), and pulmonary *Aspergillus* (176). Alternatively, rather than DC-like or macrophage-like differentiation, monocytes may remain as tissue monocytes upon extravasation (177).

CD14^+^CD16^+^ intermediate and CD16^+^ non-classical monocytes are expanded in multiple disease, infection, and inflammatory states (178). CD16^+^ monocytes “patrol” the endothelium *in vivo*, are weak phagocytes, and sense nucleic acids and viruses via TLR7 and 8 receptors (155). Additional heterogeneity has been reported within human monocytes. Tie2^+^ monocytes are associated with angiogenesis and Slan^+^CD16^+^ cells, which are also present in inflamed skin, are potent producers of TNF α, IL-1β, and IL-12 (179, 180). Monocyte-derived dermal CD14^+^ cells express IL-1α (25) have been shown to induce differentiation of follicular helper T cells (126) and provide direct B cell help (181).

### Langerhans cells

Langerhans cells are located in epidermal surfaces such as skin and are characterized by the presence of cytoplasmic organelles containing Langerin called Birbeck granules (182). The function of these organelles is unclear but their absence does not affect their capacity to process and present antigen (183). LCs form a dynamic network with adjacent keratinocytes and protrude dendrites through tight junctions to pick up antigens that have passed the stratum corneum barrier (184). The easy accessibility of LCs and their functional plasticity has generated significant interest in targeting them for vaccination strategies (185).

In the steady state, LCs are maintained independently of the bone marrow through local self-renewal (186–188). Human LCs can proliferate *in situ* and have been shown to remain donor in origin up to 10 years after limb transplant (189–191). During inflammation, LCs can be replaced by circulating precursors. The identity of the circulating LC precursor remains unclear. In mice, there appears to be two waves of replenishment with monocytes in the first wave giving rise to short-term LCs that retain some monocyte features and an as yet unknown CD34^+^ HSC-derived precursor that gives rise to long-term LCs (186, 187). In humans, CD1c^+^ DCs are able to upregulate langerin and CD1a, a phenotype resembling LCs, upon *in vitro* culture with TSLP and TGFβ or GM-CSF and BMP7, but the relevance of this to *in vivo* LC differentiation is uncertain (192, 193). Although human LCs can self-renew locally after BMT, they are replaced by donor-derived cells, even after non-myeloablative transplant conditioning (194–196).

Langerhans cells are developmentally independent of Flt3 but dependent on Csf1r. However, it is IL-34 signaling through Csf1r, rather than Csf1, that is critical for LC development and maintenance (197). IL-34 is also expressed in human skin but the dependence of human LCs on this cytokine remains untested.

#### Phenotype

Human and murine LCs are CD11c^lo^, langerin^hi^, EPCAM^+^, and also characterized by the presence of cytoplasmic Birbeck granules (198). In human, LCs are additionally CD1a^hi^ and CD1c^+^ (23, 199).

#### Homology

The homology between LCs in humans and mouse is obvious given their exclusive anatomical occupancy and shared expression of langerin, EPCAM, and presence of Birbeck granules. Comparative transcriptomic analysis of human and mouse LCs has never been performed.

#### Transcription Factors

Langerhans cell development is dependent on PU.1, Runx3, and Id2, although the latter may be dispensable for bone marrow-derived LCs (74, 188, 200, 201).

#### Function

Langerhans cells are able to induce different immune responses depending on the context. Depletion of murine LCs can either exacerbate or suppress contact hypersensitivity immune response [reviewed in Ref. (202)]. In a mouse model of graft versus host disease (GVHD), LCs neither primed CD8^+^ T cells nor programed their homing to the epidermis but were required for their effector function *in situ* (203). This is consistent with their inability to cross-present antigen *in vivo* (80, 204), although cross-presentation has been reported using *in vitro* assays (205). In mice, LCs appear to be critical for Th17 response against the yeast form of *Candida albicans* in the epidermis through engagement of Dectin-1 and their subsequent production of IL-6 (206). In humans, failure to generate effective Th17 responses (as a result of a range of mutations in, for example, IL-17RA, IL-17F, STAT1 genes) can result in chronic mucocutaneous candidiasis (CMC) (207). However, it is unclear if immunity against *Candida* infections in the skin in healthy individuals is dependent upon LCs. Notably, human LCs do not appear to express Dectin-1, which is important for *Candida* recognition (208). *In vitro* human LCs appear versatile and are capable of generating Th1, Th2 (209), Th17 (210), Th22 (211), and Treg (212) responses depending on the experimental conditions used.

### Macrophages

Macrophages are a diverse population of tissue-resident cells with roles in inflammation, tissue homeostasis, and repair. Macrophage identity and function can be influenced by three variables: (1) resident tissue environment; (2) exposure to activation signals; and (3) ontogeny (monocyte- vs. prenatal precursor-derived) [reviewed in Ref. (3)].

The nomenclature of macrophages is based upon their tissue of origin [for example, Kupffer cells (liver), osteoclasts (bone), and microglia (CNS)]. This is in recognition of the central influence of environment on their phenotype and function. Examples of these functional specializations include breakdown of RBCs (Kupffer cells and splenic macrophages), bone resorption (osteoclasts), gut peristalsis (muscularis macrophages), and neural network development and maintenance (microglia) (213–215). Although macrophages in the vast majority of tissues, except dermis and the lamina propria, are prenatally derived, their preservation into adulthood by self-renewal is variable by site and in the presence of inflammation [(15, 216, 217) and reviewed in Ref. (218)]. The relative preservation of dermal macrophages and LCs in patients lacking circulating blood monocytes and DCs due to heterozygote GATA2 and biallelic IRF8 deficiencies supports a prenatal origin of some human macrophages (59, 60).

Microarray transcriptome analysis has identified several thousand transcripts with greater than twofold difference in expression between macrophages from different sites in mice (219), supporting unique local microenvironment-related characteristics. These tissue specific transcripts are more prominent within macrophages than DCs (219) and may reflect the tissue-resident nature of macrophages. The impact and underlying mechanisms of environmental regulation on macrophages was elegantly demonstrated by the unique epigenetic modulation of macrophage in distinct tissues and the ability of macrophages from one environment to develop the characteristics of their counterparts in another tissue (220, 221).

#### Phenotype

Murine macrophages express the antigens CD11b, CD68, CSF1R, and F4/80 (215). With the exception of F4/80 which is predominantly expressed on eosinophils (222), these antigens are also expressed on human macrophages (223). Furthermore, human alveolar macrophages were shown to express many antigens, which are conserved at transcript level with murine bone marrow-derived macrophages (163).

#### Homology

Comparative analysis between human and mouse macrophage populations has been poorly studied. In skin, homologous monocyte-derived dermal macrophage populations have been identified (25) but the murine counterparts of human dermal macrophages containing melanin-granules (melanophages) remain uncertain. While a range of transcriptional analyses of human macrophage populations in health and disease have been performed, comparisons between human tissues and across species have not been rigorously undertaken (224).

#### Transcription Factors

The transcriptional requirements of murine YS-derived macrophages differ to those of HSC-derived macrophages. YS-derived microglia require PU.1 and Irf8 but are independent of Myb, Id2, Batf3, and Klf4 (2, 12, 225). Consistent with macrophage tissue specializations, additional TFs such NFATc1 and Spi-C have been shown to be required for osteoclasts and splenic and bone marrow macrophage differentiation, respectively (226–228).

#### Function

The M1/M2 paradigm has been described to model the diverse programs of macrophage activation but has largely relied on *in vitro* generated macrophages. This has provided a useful tool to examine macrophage activation in the absence of tissue-specific effects. More recently, a spectrum of responses, with M1 and M2 being two poles of a continuum that is transcriptionally apparent, were identified (229). It is unclear how closely human and murine macrophages are aligned in response to a similar range of stimuli. There are inter-species differences in the response to a single stimulus (LPS) between human and mouse *in vitro* derived macrophages; INOS transcript is preferentially induced in mouse but human macrophages characteristically upregulate CCL20, CXCL13, IL-7R, P2RX7, and STAT4 (230).

### Mononuclear phagocytes in inflammation

Classical Ly6C^hi^CX3CR1^lo^ monocytes infiltrate inflamed tissues where they can acquire either DC or macrophage properties (33, 231). This *in vivo* process (thought to be analogous to *in vitro* mo-Mac and mo-DC differentiation) can be influenced by local microbiota (97, 98, 170, 171). In infection and disease, monocyte-derived cells accumulate in greater numbers in a broad range of tissues [reviewed in Ref. (232)]. In many such models of infection, they are non-redundant and required for clearance of pathogens by promoting protective Th1 and Th17 responses (34, 233, 234). This suggests that despite shared functions with resident conventional DCs, there are important differences that require the presence of monocyte-derived cells to overcome infection. In murine experimental autoimmune encephalomyelitis, monocytes infiltrate the CNS but are not long-lived and following resolution do not contribute to the microglial pool (231). Analysis of murine Kupffer cells suggests functional heterogeneity between resident and recruited populations (235).

Snapshot analysis of inflamed human tissue similarly reveals additional subsets that are not present in health [(47, 99, 179, 236, 237) and reviewed in Ref. (31)]. These include inflammatory dendritic epidermal cells (IDECs) found in atopic dermatitis, TNF, and iNOS producing DCs (Tip DCs) and slan DCs, found in psoriasis (99, 179, 236, 237). In rheumatoid arthritis synovial fluid and malignant ascites, there is an accumulation of cells that express overlapping markers with blood CD1c^+^ DCs but additionally express CD1a, CD206, SIRPα, and CD14 (47). Monocytes can acquire DC characteristics when cultured with *ex vivo* GM-CSF-primed synovial T cells, which potentially suggests a mechanism for their generation (238). Histiocytes are pathological MPs expressing CD68 and CD163. It is unknown if these cells, often found in granulomas, arise from resident macrophages or are monocyte-derived. Further studies are required to establish the *in vivo* differentiation requirements of inflammatory MP populations and how they contribute to disease.

## Conclusion

In this review, we have discussed the parallel organization of the MPS between humans and mice. We demonstrate the use of comparative biology approaches as both a validation and discovery tool to dissect the development and functional heterogeneity of mononuclear phagocytes in a reciprocal manner across the two species. The incorporation of high-dimensional unbiased single-cell genomics and proteomics technologies will facilitate the interrogation of functionally relevant populations with indiscrete phenotypes and validate current definitions of cell-types based on limited antigen expression profile particularly during inflammation. This combined strategy will accelerate the translation of fundamental MPS biology to clinical benefit through enhanced understanding of the pathomechanisms of disease and facilitate the development of novel approaches in vaccination and cancer immunotherapy.

## Conflict of Interest Statement

The authors declare that the research was conducted in the absence of any commercial or financial relationships that could be construed as a potential conflict of interest.
